# Design and evaluation of a learning assignment in the undergraduate medical curricula on the four dimensions of care: a mixed method study

**DOI:** 10.1186/s12909-021-02681-0

**Published:** 2021-05-31

**Authors:** Jolien Pieters, Daniëlle M. L. Verstegen, Diana H. J. M. Dolmans, Franca C. Warmenhoven, Marieke H. J. van den Beuken - van Everdingen

**Affiliations:** 1 Department of Educational Development and Research, Faculty of Health, Medicine and Life Sciences, Maastricht University, Universiteitssingel 60, 6229 ER Maastricht, The Netherlands; 2 Centre of Expertise for Palliative Care, Maastricht UMC+, Maastricht, The Netherlands

**Keywords:** Chronic care, Palliative care, Undergraduate medical curricula, Authentic task, Peer feedback, Reflection, Design-based research

## Abstract

**Background:**

Chronic and palliative care are rapidly gaining importance within the physician’s range of duties. In this context, it is important to address the four dimensions of care: physical, psychological, social, and spiritual. Medical students, however, feel inadequately equipped to discuss these dimensions with the patient. To bridge this gap, a new assignment was developed and implemented, in which students talked to a chronic or palliative patient about the four dimensions of care during an internship. This study, reports the evaluation of this assignment by students and teachers using a design-based approach.

**Methods:**

Mixed methods were used, including a) student questionnaires, b) student focus groups, c) teacher interviews, and d) student’s written reflections. Two researchers performed analyses of the qualitative data from the focus groups, interviews, and written reflections using qualitative research software (ALTLAS.TI). Descriptive statistics were computed for the quantitative data using SPSS 21.0.

**Results:**

Students and teachers valued talking to an actual patient about the four dimensions of care. Reading and providing peer feedback on each other’s reports was considered valuable, especially when it came to the diversity of illnesses, the way that patients cope and communication techniques. The students considered reflection useful, especially in the group and provided it was not too frequent. All the dimensions were addressed in the interviews, however the spiritual dimension was found to be the most difficult to discuss. The analysis of the written reflections revealed an overlap between the social and spiritual dimensions. Students pay a lot of attention to the relationship between the illness and the patient’s daily life, but the reflections do often not show insight in the potential relationship between the four dimensions and decisions in patient care.

**Conclusions:**

During internships, medical students can practice talking about four dimensions of care with a chronically ill or palliative patient. Due to the format, it can be implemented across existing internships with relatively little extra time and effort. Reflection, peer feedback, and group discussion under the guidance of a teacher are important additions.

**Supplementary Information:**

The online version contains supplementary material available at 10.1186/s12909-021-02681-0.

## Background

The growing elderly population and advancing medical insights have given chronic and palliative care more prominence in the physician’s range of duties. Central to chronic and palliative care is the holistic approach. This approach concerns the four dimensions of care: physical, psychological, social and spiritual. The provision of such holistic care poses complex challenges [1]. It is important, therefore, that medical students acquire the necessary knowledge and skills before they enter the professional setting and that they learn to communicate with a patient about the four dimensions of care. Basic palliative care training to all medical and nursing students has been a long-standing credo of the palliative care community [2], and had it been implemented, the health-care professionals would be more prepared for the COVID-19 pandemic [3]. The competences that medical students need to acquire to do so have recently been set out in an educational framework [4], but prior research has shown that medical students feel ill-prepared to raise and discuss the four dimensions [5]. Their education primarily focuses on one dimension - the physical - while allowing the others to fall by the wayside.

To fill this gap, a new assignment was developed to teach students to communicate about the four dimensions of care with a patient. This assignment was implemented in an existing internship in which students encounter chronic patients on a regular basis. Addressing the four dimensions is a complex task. According to current educational principles, complex learning can be supported by an assignment that constitutes a realistic or authentic task [6]. Realistic, authentic tasks allow students to acquire the necessary knowledge, skills and attitudes integrally [7]. This, in turn, improves the transfer of the curriculum to the workplace [6]. The authenticity of the assignment in this study lay in the fact that the student was to question a real patient encountered in the professional practice about the four dimensions of care.

The principles of peer feedback and reflection also play an important role in contemporary learning. Feedback is essential for learning [8–10]; it can help students recognize their possible shortcomings in their knowledge, skills, or attitudes [8]. Students who provide peer feedback experience increased responsibility. It also initiates an active and self-managed learning process [8]. It may be a reliable assessment for professionalism and aid in the development of professional behavior [11]. At the same time, the students reflect on their own work by considering the work of others. They learn to use assessment criteria, which they can then apply to their own work [12]. The ability to reflect on one’s own performance is regarded as crucial for personal and professional development [13–16]. Reflection is an essential component in medical curricula [16]. It creates a greater understanding of both the self and the situation, so future actions can be informed by this understanding [16]. The ability to reflect on one’s own performance is regarded as crucial for personal and professional development [13]. Reflection encourages students to think more deeply about their role as a physician [13], foster professional growth and patient care skills [15, 16] . Recent research shows that written reflection about difficult patient interactions allow students to explore emotions, motivation and identity [17].

This article reports on the assignment’s design based on educational principles, its implementation and the evaluation with students and teachers. The evaluation sought to answer the following question: How do students and teachers evaluate an assignment in which students communicate with a chronically ill patient about the four (physical, psychological, social and spiritual) dimensions of care, provide each other with peer feedback and reflect?

## Methods

### Approach

A Design-Based Research (DBR) approach was used. This methodology revolves around the design of education, based on both theoretical principles or design guidelines and the input of various stakeholders. Research that uses the DBR methodology must meet the five following conditions [18]: Firstly, the intervention should be implemented in a real-life setting where learning normally takes place. In this study, we implemented the learning assignment in an internship. In this real-life setting, students encounter chronic patients and sometimes palliative care patients. Secondly, the DBR methodology requires that various stakeholders should be involved. This assignment was developed by a team of instructional designers, teachers, educational researchers, and practitioners. Thirdly, the design of the intervention has to be based on theoretical principles. This intervention is based on the principles of authentic tasks, peer feedback, and reflection. Fourthly, the DBR methodology requires continuous cycles of design, evaluation, and redesign. In this research, we evaluate the intervention to redesign it. And lastly, it should include a mixed methods evaluation. In this study, a concurrent triangulation mixed methods was applied [19], evaluation methods were a questionnaire, focus groups with students, interviews with teachers, and an analysis of reflection reports.

### Setting

The undergraduate curriculum in Medicine in the Netherlands covers 6 years divided into two parts. The first 3 years form the Bachelor’s degree and the last 3 years are the Master’s degree that consists largely of internships. At Maastricht University, the internship in Neurosciences is scheduled into the fifth year of the curriculum. It is a 20-week internship covering four medical disciplines: ophthalmology, otolaryngology, neurology and psychiatry. It allows students to encounter patients living with one or more permanent limitations as a consequence of a chronic or incurable illness. The students meet every week, during in-house sessions, to discuss theory and experiences. The new assignment was placed on the agenda for three of these sessions. Every 2 weeks, there is a new group of students starting this internship.

### Assignment

#### General description

**Fig. 1:**
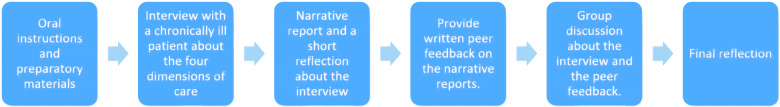
The assignment

First, students were asked to study the preparatory materials consisting of literature on the four dimensions of care and a topic list with questions that students may use during the interview. Either alone or together with a fellow student, the student then interviewed a patient with a chronic condition, focusing on the four dimensions of care. Next, the student was asked to write a narrative report and a brief reflection on the interview, describing the way the patient dealt with the condition or illness related to each of the four dimensions. Each student discussed their interview with the group in the in-house session. They also provided peer feedback on the reflection reports of two fellow students. At the end of their internship, students wrote a final reflection. The interview with the patient could take place in either a clinical or home setting. As for the interviewee, the student asked a patient they had met during their internship for an interview (see Fig. 1). The assignment was designed on the basis of three educational principles: it was an authentic assignment, in which the students provided peer feedback and reflected on their own experiences.

#### Authentic assignment

Each student interviewed a real patient they had met during their internship. The students were encouraged to conduct the interview in such a way that optimal attention was paid to the four dimensions of care. To help them, the students received a list of example questions, such as “*What impact do you believe your illness has on your future?”*

#### Peer feedback

Students gave peer feedback in the form of assessing two narratives and reflection reports from fellow students and received peer feedback from two fellow students themselves. Peer feedback was discussed in the weekly in-house sessions that are organized as part of the internship.

#### Reflection

Students were encouraged to reflect on their own interview and their personal values and beliefs with regards to the four dimensions of care, in a reflection report. The agendas for the weekly in-house sessions included the necessary time for subsequent discussion and joint reflection, under the supervision of a teacher. By the end of their internship, they wrote a final reflection report.

### Participants

A total of 30 students completed the questionnaire and participated in the focus groups, 24 of whom also gave permission for analysis of their reflection reports. A total of 50 students had been invited to participate, 12 of whom declined, and 8 were excluded when it became clear that they came from a different stream in the Bachelor degree which included a similar (but now cancelled) assignment. The 30 participating students were divided into 4 focus groups. Their average age was 23 (SD = 1.46), and 19 students were female. In addition, three participating teachers were interviewed.

### Instruments

**Fig. 2:**
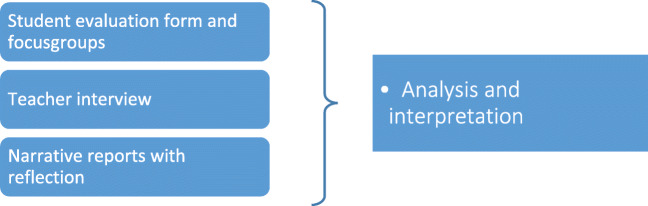
The applied concurrent triangulation mixed methods

This study used concurrent triangulation mixed methods (see Fig. 2) to validate the findings generated by each method through evidence produced by the other methods [20]. Two groups of stakeholders were asked to participate in the evaluation: students and teachers. The students were asked to complete a short questionnaire and participate in the focus group (FG) in which the assignment was evaluated extensively. The teachers were interviewed. Furthermore, the students’ reflection reports were examined in order to analyse the topics discussed.

#### Student evaluation form

Students completed a questionnaire with 17 items about the assignment on a five-point Likert scale (range: 1 = totally disagree; 5 = totally agree). The questionnaire was developed by the researchers JP and DV. The questionnaire was developed by the researchers JP and DV. The items were based on the educational principles used in this study (authentic learning, peer feedback, and reflection). After discussing with the research team, some of the items were reworded. A question regarding the final reflection was removed from the questionnaire; the students could not answer this question because the questionnaire preceded their final evaluation. The questionnaire was divided into four parts: authentic task, the four dimensions, peer feedback, and reflection. It also included two open questions: “What did you find most valuable about the assignment?” and “What did you find least valuable about the assignment?”

#### Student focus group

In the focus groups, the students discussed the assignment and supporting resources using a semi-structured interview guide (see Additional file 1). The questions concerned the students’ experiences with regards to the assignment, such as “How did you experience interviewing a real patient about the four dimensions, what worked well and why, what worked less well and why?”, “How did you experience the group reflection/peer support?” and “How did you experience to be a provider of peer feedback?”

#### Teacher interviews

Semi-structured interviews (see Additional file 1) were used for the teacher evaluations. The questions concerned the teachers’ experiences with regards to the assignment, such as “How did the teachers experience the communication assignment?” and “Did the students learn from these assignments?”

#### Reflection reports

The researchers inspected the reflection reports written by the students about their interviews. The analysis focused on the subjects the students had included in their reports, for example, to what extent students had addressed the four dimensions of care. These analyses were only used for the study and not for the assessment of the student.

### Procedure

In the first in-house session, the assignment was explained and the study was introduced to the students. The FG meetings were held directly after the last in-house session. A week before the FG meetings, the students were asked to participate by an email sent from the first researcher (JP). The information letter was added to this email. Prior to the study, the participating students gave their written consent. Patients gave their consent to the interview with the students. The students anonymized the patients in their reports. Thereafter, the student reports were anonymized for the researchers. At the beginning of their FG meeting, the students were asked to complete the questionnaire before the actual discussion began. Each FG consisted of 6–8 students who met for a maximum of 45 min. The students were also asked whether the researchers were allowed to inspect their reflection reports. Their permission was also obtained through the informed consent form. The teachers were evaluated through individual interviews. They were invited by an email that included the information letter. The interviews took no more than 45 min.

### Analysis

For the questionnaire, SPSS 21.0 was used to obtain descriptive results. The mean scores per item (*N* = 17) were computed across all students. For the qualitative part, the data analysis was supported by Atlast.ti. The focus group meetings, interviews and reflection reports were analysed based on a thematic analysis, taking into account the step-by-step plan of Brooks and colleagues [21]. Researchers JP and DV familiarised themselves with the data. They were both involved in the focus group meetings and read the transcripts. Preliminary coding of the data started with determining the a priori themes. These themes were based on the research question (authentic learning tasks, the four dimensions, peer feedback, and reflection). Two focus group transcripts were read and independently coded by researchers JP and DV. They discussed their findings, divided the themes into meaningful clusters and created an initial coding template. This initial template was subsequently applied to the last focus group transcript and the three interview transcripts, independently by researchers JP and DV. The researchers discussed and finalised the template and applied it to the full data set. The reflection reports were then coded with the same coding scheme.

### Reflexivity

This assignment was developed by a multidisciplinary group that included teachers, educational theorists, researchers and physicians. Researchers JP and DV coded and analysed the data. JP is a doctoral student with a background in psychology and educational research. DV is an educational theorist with extensive experience in educational research. Due to the background of these two researchers, the analysis focused primarily on the students’ learning process and the design principles. Both researchers co-designed the assignment. Researchers DD, MvdB and FW contributed ideas and helped shape the analysis. DD is an educational scientist and has expertise in instructional design. MvdB is an internist and medical consultant palliative care. FW is a researcher and former general practitioner with expertise in both palliative care as well as medical education. The different perspectives have enhanced the strength of this paper. But the disparate backgrounds also may have influenced our views on educational principles and the assignment itself.

## Results

### Evaluation form

The students scored the assignment on 17 items (Table 1). The scores varied between 3.00 (SD = 0.76) and 4.63 (SD = 0.49) (scale 1–5).

**Table 1:** Students’ views on the new assignment (*N* = 30) (Scale 1–5; 1 = Totally disagree; 5 = Totally agree)

	Question	Mean score (SD)
***Authentic task***	The assignment gave me more insight into the influence of the four dimensions on the care (to be) provided.	3.27 (0.77)
	The content of the assignment was in keeping with my prior knowledge and skills.	4.03 (0.67)
	The assignment encouraged me to think about my future role as a physician.	3.50 (0.82)
	I found it instructive to interview a real patient.	3.80 (0.93)
***Four dimensions***	I dared to discuss the four dimensions of care with the patient.	4.20 (0.55)
	I talked to the patient about the physical dimension.	4.40 (0.86)
	I talked to the patient about the psychological dimension.	4.57 (0.56)
	I talked to the patient about the social dimension.	4.63 (0.49)
	I talked to the patient about the spiritual dimension.	3.43 (1.19)
***Peer feedback***	I found it instructive to give peer feedback.	3.13 (0.68)
	I found it instructive to receive peer feedback.	3.40 (0.72)
	I trust the peer feedback I received from my fellow students.	4.27 (0.69)
	I found it useful to read the reports by my fellow students.	3.73 (0.64)
***Reflection***	I found it instructive to write the interview report.	3.20 (0.81)
	I found it instructive to discuss my interview with the patient in the group.	4.00 (0.70)
	I learned a lot from the “4D interview” assignment.	3.40 (0.81)

The average scores for the items about the authenticity of the assignment were 3.50 or higher, with the exception of the item about the influence of the four dimensions on care, which scored an average of 3.27 (SD = 0.77). The students specified that they discussed the four dimensions during the interview. The physical, psychological, and social dimensions all scored higher than 4.00; only the spiritual dimension received a lower average score of 3.43 (SD = 1.19).

The scores for providing and receiving peer feedback ranged from 3.13 (SD = 0.68) to 3.40 (SD = 0.71). Students trusted the peer feedback they received, with an average score of 4.27 (SD = 0.69). The scores for reflection varied between 3.20 (SD = 0.81) and 4.00 (SD = 0.70). The learning effect of the assignment scored 3.40 (SD = 0.81).

In the open questions, students stated that they found it most valuable to have more time with a patient than they have as a (trainee) practitioner. They considered the influence of the illness on the patient’s daily life the most informative. The students valued that they had gained more knowledge about the four dimensions of care. They considered reflection valuable and also discussing peer feedback in the group. Providing written peer feedback was found least valuable, as it was time-consuming and did not render any new insights.

### Focus group meetings with students and interviews with teachers

#### Authentic task

Interviewing a real patient was perceived as beneficial by both the students and teachers. Students found it interesting to hear the patient’s story from the patient themselves or, in some cases, from the patient’s partner. A number of students explicitly stated that they had discovered or tried new communication strategies. The students believed that they managed to discuss all four dimensions using the topic list they received. The spiritual dimension was felt to be the most difficult to question. The students believed that there was quite some overlap between dimensions, especially where the social dimension was concerned. Some students stated that they were not used to talking about the spiritual dimension or that the patient did not really want to talk about it. According to the teachers, however, the students addressed all of the four dimensions, but had as yet not dared to probe and look for depth. Teachers recognise this in the way students reported on the patients’ coping with their illness: almost all of the patients turned out to have a positive outlook on life; while some said their condition did not cause them much discomfort. This led the teachers to conclude that the students had not gone into enough depth. Students, on the other hand, claimed that this was a consequence of selection. Most had opted for patients with an open and easy demeanour and, thus, for patients with a positive outlook on life.

*“This makes you genuinely curious. If you have someone sitting opposite you, then, well, you are interested in that story. And if a simulation patient then says something, well, then you do it because you have to.” - Student, focus group 1.*

*“I did notice that some students found it difficult to ask probing questions about certain things because then, because they thought, well, that is too sensitive, but that they then sometimes also had the idea of ​​yes, that they would er … that it would be unprofessional to ask about certain matters.” - Teacher 2.*

*“That they almost always say, like: ‘Well, er… those patients, what strong people they are, how well they deal with those… with those complaints’. I think that is what many do. And that disappoints me. That, that actually shows me that they don’t adequately probe and seek depth.” - Teacher 1.*

*“You do select patients, naturally. You do not go for the most … say the most grumpy, the one you have no connection with at all, you will not interview that one. For … so you pick people who are a bit up your alley, whom you believe: oh, this is one I can ask things. Not someone who has just been diagnosed and is in a very foul mood.” - Student, focus group 4.*

#### Peer feedback

By reading each other’s reflection reports, the students saw more diversity in conditions, the influence on patients’ lives and the ways in which they coped. A number of students stated that reading the others’ reflection reports had taught them more about interview techniques. Both teachers and students found the group discussion/reflection the most valuable. The students enjoyed being able to ask each other questions, in terms of both content and the way in which the other person had conducted their interview. The teachers said that the group discussion allowed them to ask questions to encourage the students to reflect more deeply*.* Students found the obligation to provide written peer feedback unnecessarily time-consuming.

*“Yes, by reading it you very much come across the diversity of chronic conditions. You also talk about it in the group, but you’re just a little more aware of it when you actually have to read a report that you, say, like, have to reflect on.” - Student, focus group 2.*

*“How other people experienced the interview. And they see or often face other bottlenecks in such an interview than the ones you may have encountered. And that’s interesting to talk about.” - Student, focus group 3.*

*Reflection.*

Both the students and teachers considered reflection a logical and important part of this assignment. The students believed that reflecting after the interview makes sense, provided it was not required too often. The group reflection was found to be the most valuable4. The teacher stated that the students mainly reflected on surprising scenarios, particularly on the greater than expected influence of the illness on the patient’s life and the way in which the patient coped with the illness in their daily life. The students reflected far less on the influence of the four dimensions on the care (to be) provided. Only a few students made the connection between the two.

*“This [reflection] forces you to run through everything once again and categorise it a bit, like, what have I actually questioned until the last detail.” - Student, focus group 2.*

*“On the unexpected findings, let me put it that way. Things they … so they reflect on things they have heard which they realise they would not have heard in the doctor’s office.” - Teacher 1.*

*“Yes, I did think it did give, at least in the case of my patient, a clearer idea of ​​what the patient’s needs were with regards to the choice of treatment and so on. Something you normally skim over in a flash, so to speak.” – Student, focus group 1.*

*‘[Reflecting] feels a bit more like, say, a mere obligation now. Like: here we go. My response was: here we go again. Like I have to write again and you have to reflect on yourself and on your … and on other conversations, and then a final reflection and … that you are just reflecting to reflect, that is how it feels.” - Student, focus group 4.*

### Written reflections

All four dimensions of care were addressed in most reports, but the physical and social dimensions were typically the most extensively discussed, usually related to the impact of the illness on the patient’s life. The spiritual dimension was least explored, although many students realised that, for many patients, meaning and purpose were bound up with the social dimension: the importance of being able to undertake activities with family or friends. Otherwise, the reflections mainly focused on the influence of the patient’s illness on daily life, coping and experiences with the care system. Only a few made a link between the four dimensions, care provision and their own role as a (future) physician. The reports show that most students stuck to the topic list as the reports had the same structure.

*“Living with the uncertainty that is so inherent in the illness has the same effect on life as many chronic illnesses. I think that this is the most important point that can be concluded from this case; in the medical world, we tend to quickly focus on the physical limitations and somatic aspects of a chronic condition, and in a mild case as described above, quickly assume that the effect on the patient’s life must be minimal as well.” - Student 2C, quote from report.*

*“Reflecting on this interview, I can conclude for myself that I now go into the consultation hour very differently, for I can indeed mean something to these patients by, among other things, also paying attention to the emotional experience that the patient is going through.” - Student 2 M, quote from report.*

*“I found it quite an experience to meet a patient I saw in hospital at home for a change. You immediately become aware that it creates a completely different atmosphere and also feels less like a patient-doctor relationship.” - Student 2D, quote from report.*

## Discussion

This assignment taught students to talk to patients about the four dimensions of care. In the focus group meetings, the students stated that they found the authenticity of the assignment, i.e. interviewing a real patient, instructive. The questionnaire and reflection reports confirmed this. Reading and providing peer feedback on each other’s reports was considered valuable, especially when it came to the diversity of illnesses, the way that patients cope and communication techniques used in the interviews. The students considered reflection useful, especially in the group and provided that it was not too frequent.

Looking at the various components of the assignment, some aspects stand out**.** Firstly, in the focus group meetings the students said that they were generally quite satisfied with the way they conducted their interviews about the four dimensions, although they did find the spiritual dimension the most difficult to discuss. Other findings confirmed this: the spiritual dimension scored lowest in the questionnaire and was the least discussed dimension in the reflection reports. This corresponds to recent research that showed that also in the consultation room the spiritual dimension is the least discussed and it is experienced as the most difficult dimension. Both professionals and students attributed this to various obstacles such as too little time, discomfort and a lack of training [5, 22]. Yet, medical students underline the importance of spiritual needs [5, 23] and the skills to discuss meaning and purpose can be taught and acquired [22]. In this study, students expressed that the difficulty in discussing the spiritual dimension lay in the fact that it is largely interwoven in the psychological and social dimensions. They were also not used to talking about it. The students did ask what the patient considered important and what they felt to be ‘a good day’, as suggested in the topic list. The teachers believed that students did not always scratch below the surface and asked shallow questions. The students did not see it this way: they believed that the patients they interviewed managed to cope well with their illnesses so that there was not much depth to go into. It is indeed likely that the students selected patients with a (relatively) positive outlook on life and good coping mechanisms. However, it might also be that students are as yet not fully equipped to recognise when and how they should go into detail, and therefore require further training. Maybe they will be able to discuss the spiritual dimension in more depth when they learn more about it beforehand, i.e. about what exactly constitutes this dimension and how it is related with the other dimensions.

 Secondly, there was a small discrepancy between the findings from the focus groups and the questionnaire when it came to receiving and providing peer feedback. In the focus groups, the students rated that providing peer feedback is more valuable than receiving it. This, however, did not emerge from the questionnaire. The students filled in the questionnaire directly before starting the focus groups. During the focus groups was a more in-depth discussion regarding this topic, which may have resulted in students changing their views. Therefore it is most likely that the focus groups results reflect are a more representative view. This would also be in line with the literature that states that providing feedback leads to greater improvements than receiving feedback [24–26]. By providing peer feedback, students take an active, reflective role in learning [12]. Furthermore, students develop problem-solving skills [25] and learn the use the assessment criteria, which they use to improve their own work [25].

Thirdly, it appeared that the students found it beneficial to reflect, provided they did not have to do it too often. Although reflection is a useful tool, too much reflection creates resistance among students [16]. Furthermore, it can fail to achieve its objectives, when students have been conditioned to follow prescribed thought steps rather than engaging in truly reflective behavior [27].

Finally, it was striking that students pay a lot of attention to the relationship between the illness and the patient’s daily life, whereas the relationship between the four dimensions and care remained unclear for most of them. This came to the fore in both the focus groups and the questionnaire. We did see a small group of students make this link in the reflection reports. They discussed how the dimensions might influence the patient’s care plan. To safeguard the transfer to practice, students will have to learn to make this link. As future medical professionals, they should not only be able to discuss the four dimensions with the patient, but also translate the outcomes of the discussion into the care plan. Several factors may play a role as to why the students did not make this link. To begin with, the students stuck to the topic list, which did not include an explicit question concerning the four dimensions and care. Secondly, it may also be that the teachers placed an emphasis on the way illnesses affect the patients’ daily life, but put little focus on how they affect the care (to be) provided.

This study had some strengths and limitations. The implementation of the assignment in practice is a strong point: it shows that attention for the four dimensions of care can be integrated in internships. This also means, however, that it was examined in a specific context and that the results are affected by the design of this specific curriculum in a problem-based education system [28, 29]. The students involved in this study were already familiar with discussions, peer feedback and reflecting. The effectiveness of this assignment in a different context remains to be investigated. Secondly*,* we used triangulation of different data sources: the results were based on the experiences of the students, teachers and the inspection of reflection reports*.* The researchers did, however, not observe the interviews between the students and patients themselves. Thirdly, students interviewed chronically ill rather than palliative patients. The advantage of this is that students meet many chronically ill patients in this internship and it was not difficult for them to find patients that were willing to be interviewed. This means, however, that most students did not talk to palliative care patients. Thirdly, this assignment comprised one extensive assignment. Proper education in chronic and palliative care requires that the associated topics be integrated into the curriculum and discussed when relevant [30]. In order to prevent the training from becoming too generalised [31], a learning programme should be developed*.* For example, prior to this assignment, students could learn more about the four dimensions, and the spiritual dimension in particular. A subsequent assignment could then focus on teaching the students to make a clearer link between care and the four dimensions. Future research should focus on the longitudinal integration and evaluation of learning activities focused on communicating with patients about the four dimensions of care in the entire curriculum.

This study contributed to the knowledge about integrating attention for palliative care in the undergraduate curriculum. Due to the format, this assignment can be implemented across existing internships with relatively little extra time and effort, taking into account the educational principles and a number of key elements and. The first is that enough time is allowed to discuss the assignment in the groups. Both the students and teachers considered the group discussions to be very valuable. There should also be a clear topic list with possible questions to ask the patient, especially if it is the first time that the students talk to patients about the four dimensions of care. It provides a foothold and ensures that the students dare to discuss the dimensions. Furthermore, it is important that teachers take their own educational institution into account and consider to what extent students are familiar with the four dimensions, reflection and peer feedback.

## Supplementary Information

**Additional file 1.**

## Data Availability

The dataset is available from the corresponding author on reasonable request.
