# Supplementary material for: Design and evaluation of a learning assignment in the undergraduate medical curricula on the four dimensions of care: a mixed method study
Source: BMC Med Educ. 2021 May 31;21:309. doi: 10.1186/s12909-021-02681-0 (PMC8165949; doi:10.1186/s12909-021-02681-0)
Supplement: Supplementary file 1 — Additional file 1. [file 12909_2021_2681_MOESM1_ESM.docx]

Design and evaluation of a learning assignment in the undergraduate medical curricula on the four dimensions of care: A mixed method study

**Educational intervention in DBR setting**

Jolien Pieters, MSc, Daniëlle M.L. Verstegen PhD, Diana H.J.M. Dolmans, PhD, Franca C. Warmenhoven, PhD, MD, Marieke H.J. van den Beuken van Everdingen, PhD, MD.

Department of Educational Development and Research (J.P., D.D., D.V., F.W.),

Faculty of Health, Medicine and Life Sciences, Maastricht University, Maastricht; and

Centre of Expertise for Palliative Care (M.v.d.B), Maastricht UMC+, Maastricht, the

Netherlands.

Please address correspondence to: Jolien Pieters, MSc,

Universiteitssingel 60, 6229 ER, Maastricht, the Netherlands.

Tel: +31 43 388 5656.

Email: j.pieters@maastrichtuniversity.nl

**Appendix: interview guide**

**Semi-structured interview guide for students:**

Introduction: Please introduce yourself (name; age)

*Transition question:*

1) How did you experience the assignment around the four dimensions and the associated education in general?

*Thank you. The assignment has been designed based on certain educational ideas and principles. I would like to elaborate on this with you.*

1) How did you experience interviewing a real patient about the four dimensions of care? What worked well and why? What worked less well and why? What did you learn? (Authentic learning task)

2) How did you experience writing the narrative report and your personal reflection on this conversation? What was instructive / less instructive and why? (Reflection)

3) How did you experience GIVING peer feedback? What was instructive / less instructive and why? (Peer feedback)

4) How did you experience RECEIVING peer feedback? What was instructive / less instructive and why? (Peer feedback)

5) How did you experience the group reflection / Intervision? (Reflection)

6) How did you experience the final reflection? (Reflection)

*Finally*,

1) Are there other things that are relevant to discuss, and that have not yet been addressed?

*Thanks*.

**Semi-structured interview guide for teachers:**

Introduction:

- Introduce yourself (name; function)

Transition question.

1) How did you experience the assignment around the four dimensions and the associated education in general?

*Thank you. The assignment has been designed based on certain educational ideas and principles. I would like to elaborate on this with you.*

2) What is your impression of the conversations that students have had with a real patient about the four dimensions of care? To what extent were the four dimensions covered in the individual interview reports? What worked well and why? What worked less well and why? What do you think that students have learned from it? (Authentic learning task)

3) What did you think of the depth of the students' individual reflection reports? To what extent did you see a profound critical dialogue in which the student reflects and comes up with alternative approaches? (Reflection)

4) How did you experience the group reflection / intervision? To what extent have the four dimensions been discussed and how profound was the d? (Reflection)

5) How was providing the peer feedback experienced? (Peer feedback)

6) How was accepting the peer feedback perceived? (Peer feedback)

Finally,

7) Are there other things that are relevant to discuss, and that have not yet been addressed?

*Thanks.*
